# The Expression of Testin, Ki-67 and p16 in Cervical Cancer Diagnostics

**DOI:** 10.3390/cimb45010032

**Published:** 2023-01-05

**Authors:** Aneta Popiel-Kopaczyk, Jedrzej Grzegrzolka, Aleksandra Piotrowska, Mateusz Olbromski, Beata Smolarz, Hanna Romanowicz, Agnieszka Rusak, Monika Mrozowska, Piotr Dziegiel, Marzenna Podhorska-Okolow, Christopher Kobierzycki

**Affiliations:** 1 Division of Histology and Embryology, Department of Human Morphology and Embryology, Wroclaw Medical University, 50-367 Wroclaw, Poland; 2 Department of Pathology, Polish Mother’s Memorial Hospital Research Institute, 93-338 Lodz, Poland; 3 Department of Physiotherapy, University School of Physical Education, 51-612 Wroclaw, Poland; 4 Division of Ultrastructural Research, Wroclaw Medical University, 50-368 Wroclaw, Poland

**Keywords:** TES protein, p16 protein, antigen Ki-67, cervical cancer, immunohistochemistry

## Abstract

Testin is a protein expressed in normal human tissues, being responsible, with other cytoskeleton proteins, for the proper functioning of cell–cell junction areas and focal adhesion plaques. It takes part in the regulation of actin filament changes during cell spreading and motility. Loss of heterozygosity in the testin-encoding gene results in altered protein expression in many malignancies, as partly described for cervical cancer. The aim of our study was the assessment of the immunohistochemical (IHC) expression of testin in cervical cancer and its analysis in regard to clinical data as well the expression of the Ki-67 antigen and p16 protein. Moreover, testin expression was assessed by Western blot (WB) in commercially available cell lines. The IHC analysis disclosed that the expression of testin inversely correlated with p16 (r = −0.2104, *p* < 0.0465) and Ki-67 expression (r = −0.2359, *p* < 0.0278). Moreover, weaker testin expression was observed in cancer cases vs. control ones (*p* < 0.0113). The WB analysis of testin expression in the cervical cancer cell lines corresponded to the IHC results and showed a weaker expression compared to that in the control cell line. When we compared the expression of testin in cervical cancer cell lines, we found a weaker expression in HPV-negative cell lines. In summary, we found that the intensity of testin expression and the number of positive cells inversely correlated with the expression of Ki-67 (a marker of proliferation) and p16 (a marker of cell cycle dysregulation). This study shows that the combined assessment of testin, Ki-67 and p16 expression may improve cervical cancer diagnostics.

## 1. Introduction

Cervical cancer is one of the most common cancers worldwide in women, with almost 350,000 deaths noted in 2020 [1]. Over 85% of cervical cancer deaths occurred in low and middle-income countries. In South Africa and South America countries, cervical cancer is the leading cause of death in women. Approximately, only 66.3% of diagnosed patient will survive 5 years or more. Due to the unsatisfactory prophylaxis and ineffective screening, the incidence of cervical cancer is still increasing in developing countries [2].

The main identified risk factor for cervical cancer is persistent HPV (human papilloma virus) infection, which induced the development of low- and subsequently high-grade cervical squamous intraepithelial lesions (LSIL and HSIL). Imbalances and instabilities caused by the introduction of various HPV-derived oncogenic factors into the host genome result in long-term tumor progression, but the outcomes depend on the specific HPV subtypes, mostly the high-risk subtypes (HR-HPV) [3]. Currently, 216 HPV subtypes were identified; HR-HPV 16 is responsible for more than 50% of cancer cases, while HR-HPV 18 is responsible for up to 16.5% of cancer cases [4]. Presently, the performed studies focus on seeking additional factors such as genetic and epigenetic triggers needed for the promotion and progression of cancerogenesis in patients with persistent HR-HPV infection [5,6,7,8]. Epigenetic changes are modifications of the DNA in a genome that regulate whether genes are turned off without changing the sequence of the DNA [9]. They include DNA methylation, histone modification and miRNA silencing [10].

In 2014, the estimated HPV vaccination rates in young women were around 30% in developed countries but less than 3% in least-developed countries. This clearly supports the World Health Organization goals for cervical cancer elimination, which primarily should be focused on prevention, screening and treatment of preinvasive and invasive forms of cervical cancer in low- and middle-income countries [11].

Testin is a protein encoded by the TES gene located in the fragile chromosomal region FRA7G at 7q31.1/2. In this locus, loss of heterozygosity induces the development of many malignancies as this region contains putative tumor suppressor genes such as caveolin-1 and TES [12,13]. In loss of heterozygosity, one or two alleles of the same gene are lost, so suppressor genes are inactivated. This is one of the mechanisms that decrease testin expression in cancer cells. The second mechanism leading to the loss of testin expression is associated with hypermethylation of CpG islands [14,15].

Testin is also expressed in normal human tissues. It is localized along actin stress fibers in cell–cell junction areas and in focal adhesion plaques where it can interact with other cytoskeleton-associated proteins such as talin (TLN), actin (ACT), vasodilator-stimulated phosphoprotein (VASP) and mammalian Ena homolog (Mena) [16,17,18]. Testin can take part in the regulation of actin filament changes during cell spreading and motility. The structure of testin includes three C-terminal LIM domains which have three zinc-binding domains that are responsible for protein–protein interactions and coordinate intra- and extracellular connections [19,20]. The NH-2 terminal of the TES protein can bind to its COOH- terminus. Therefore Boyan K. Garvalov et al. proposed that TES might have two conformational states: open and closed [17]. Zhong et al. confirmed that the NH-2 terminus binds to the third LIM domain but additionally found different cellular localizations of the protein. Besides the well-known localizations such as focal adhesions and cell junctions, they found the testin protein in nucleoli and the endoplasmic reticulum. This means that testin, just like other LIM protein members, is able to shuttle between the cytoplasmic and the nuclear compartments of the cell to influence gene expression [21,22,23]. The altered expression of the testin protein was described in many malignancies such as breast, colorectal, endometrial, gastric, head and neck, ovarian and prostate cancers as well in leukemia [24,25,26,27,28,29,30,31]. To date, the expression of testin was disclosed only in two cervical cancer studies [32,33].

The aim of our study was the assessment of the immunohistochemical (IHC) expression of testin in cervical cancer and its analysis in regard to clinical data as well the expression of the Ki-67 antigen and the p16 protein. Moreover, testin expression was assessed by Western blot (WB) on commercially available cell lines.

## 2. Materials and Methods

The study was approved by the Ethics Commission at the Wroclaw Medical University (approval no. 412/2019). Patient consent was waived due to the use of anonymized archival material only, which in no way influenced the diagnostic and therapeutic process.

A total of 91 cervical cancer samples were collected from women treated in the Polish Mother’s Memorial Hospital in Lodz between 2011 and 2017. The control group consisted of 92 normal tissue cases from patients who underwent total hysterectomy due to uterine leiomyomas in the same hospital. The characterization of the study group is described in Table 1.

### 2.1. Preparation of Tissue Microarrays (TMAs)

Hematoxylin-and-eosin-stained (HE) 6 μm thick paraffin sections were prepared to verify the histopathological diagnosis and assess the suitability of the samples for further analysis. In short, slides were scanned utilizing the histologic scanner Pannoramic MIDI (3DHistech, Sysmex Suisse AG, Horgen, Switzerland). The scans were then examined by two independent pathologists to select and electronically label the areas of cervical cancer cells. Then, for TMA construction, from the corresponding paraffin donor blocks, triplicate tissue core punches (2 mm) for every case were obtained (TMA Grand Master; 3DHistech). Normal cervical tissues were used as the control group.

### 2.2. Immunohistochemistry (IHC)

The immunohistochemical reactions were performed on 4 μm paraffin sections obtained from the TMA blocks using an automated staining platform, Autostainer Link48 (Dako, Glostrup, Denmark). At the beginning, deparaffinization, rehydration and antigen retrieval were performed using the EnVision FLEX Target Retrieval Solution (97 °C, 20 min; pH 6 for Ki-67 and pH 9 for p16 and testin) in PT-Link. The activity of endogenous peroxidase was blocked by 5 min exposure to Peroxidase-Blocking Reagent (Dako). The monoclonal mouse antibodies anti-p16 (1:100 + linker, 550834, BP Pharmingen, San Diego, CA, USA) and anti-Ki-67 (ready to use, IR626, Dako) and the polyclonal rabbit anti-testin (1:400, NBP1-87987, Novus Biologicals, Centennial, CO, USA) antibody were used as the primary antibodies (20 min. incubation) followed by incubation with secondary antibodies conjugated with horseradish peroxidase (EnVision™ FLEX/HRP—20 min. incubation). After the incubation, 3,3′-diaminobenzidine (DAB) was used as the peroxidase substrate, and the sections were incubated for 10 min. At the end, all sections were counterstained with EnVision FLEX Hematoxylin (Dako) for 5 min. Dehydration in graded ethanol concentrations (70%, 96%, absolute) and in xylene were the last steps, and then all slides were covered with coverslips in SUB-X Mounting Medium using a coverslipper.

### 2.3. Evaluation of the IHC Reactions

The slides were scanned with the histologic scanner Pannoramic MIDI (3DHistech). The expression was nuclear for Ki-67 and cytoplasmic for p16 and testin. The reactions were evaluated (Ki-67) with the use of Quant Center Software (3DHistech) under the researchers’ supervision. For every case, three TMA cores were quantified by the algorithm SCORE (range = 0–8), and the final result was an average count. Two parameters were used to evaluate the p16 antigen: percentage of p16-positive cells and reaction intensity. The percentage of positive cells was evaluated in the highest expression area (“hot spot”) and graded as follows: grade 0 when no cells were stained, positive cells >0–5% (grade 1), positive cells >5–25% (grade 2), positive cells >25% (grade 3). The intensity of the reaction was scored as negative (0), weak (1), moderate (2) and strong (3). The reaction was considered positive when nuclear or nuclear and cytoplasmic, strong and diffuse p16 expression was observed starting from the basal cell layer of the epithelium. The negative p16 expression was assessed when a non-specific pattern, or focal, wispy, small clusters of cells and complete lack of staining were observed. The expression of testin was assessed with the use of Pannoramic Viewer Digital image. The analysis was carried out using Immunoreactive Scale (IRS) by Remmele and Stegner, as shown in Table 2. Testin cytoplasmatic expression was assessed by using the Remmele–Stegner immunoreactive score [34]. This scale uses the percentage of positively stained cells (A) and the staining intensity of the reaction (B). The final result is the product of these two parameters (A × B).

### 2.4. Cell Lines

The human cervical cancer cells SiHa and C-33 A were cultured in Eagle’s minimum essential medium (EMEM, Lonza, Basel, Switzerland) supplemented with 1% sodium pirogronate and non-essential amino acids (Sigma-Aldrich, St. Louis, MO, USA). HeLa cells were cultured in EMEM (Lonza). The human epidermal keratinocytes HaCaT (DKFZ, Heidelberg, Germany) cell line was cultured in DMEM medium (Lonza) [35]. All media contained 1% L-glutamine, a penicillin/streptomycin solution and 10% FBS (fetal bovine serum) (Sigma-Aldrich). The cell cultures were maintained at 37 °C/5% CO2 and 95% humidity. The medium was changed twice a week, and the cells were passaged with a trypsin/EDTA solution (Sigma-Aldrich) when confluency reached about 70%.

### 2.5. Western Blot

The Western blot technique was used to determine testin expression in cervical cancer cell lines. Whole protein lysates were obtained by using the CelLytic TM MT Cell Lysis Reagent (Sigma Aldrich) with the addition of a cocktail of inhibitors (Sigma-Aldrich), 250 U of benzonase (Merck Millipore, USA) and 2 mM PMSF. The protein lysates were mixed with 4xSDS-PAGE gel loading buffer (200 mM Tris-HCl—pH 6.8, 400 mM DTT, 8% SDS, 0.4% bromophenol blue, 40% glycerol), loaded on a 10% acrylamide gel and separated by SDS-PAGE under reducing conditions, then finally transferred onto a PVDF membrane in the XCell SureLockTM Mini Gel Electrophoresis System (Life Technologies, Carlsbad, USA, USA). After protein transfer, the membranes were incubated in a blocker solution (4% BSA in TBST buffer) for 1 h at RT, followed by overnight incubation at 4 °C with the anti-testin monoclonal mouse antibody, diluted 1:500. The next step included washing the membranes with TBST buffer and incubating them for 1 h at RT with secondary donkey anti-rabbit antibodies conjugated with HRP, diluted 1:3000 (Jacksons ImmunoResearch, Mill Valley, CA, USA). Afterwards, the membranes were rinsed and treated with the Immun-Star HRP Chemiluminescent Kit (Bio-Rad, Hercules, CA, USA). Rabbit anti-human β-tubulin antibodies (2128, CellSignaling, Danvers, MA, USA) diluted 1:1000 were used as an internal control. The WB results were analyzed in the ChemiDoc MP System (Bio-Rad).

### 2.6. Statistical Analysis

All data were analyzed with GraphPad Prism 5.0 software using Spearman correlation, Kruskal–Wallis and Mann–Whitney tests. The analysis of the normal distribution of the obtained data was made by the Kolmogorov–Smirnov test. In all analyzed cases, *p*-values < 0.05 were considered statistically significant.

## 3. Results

### 3.1. IHC on Tissue Samples

**Figure 1:**
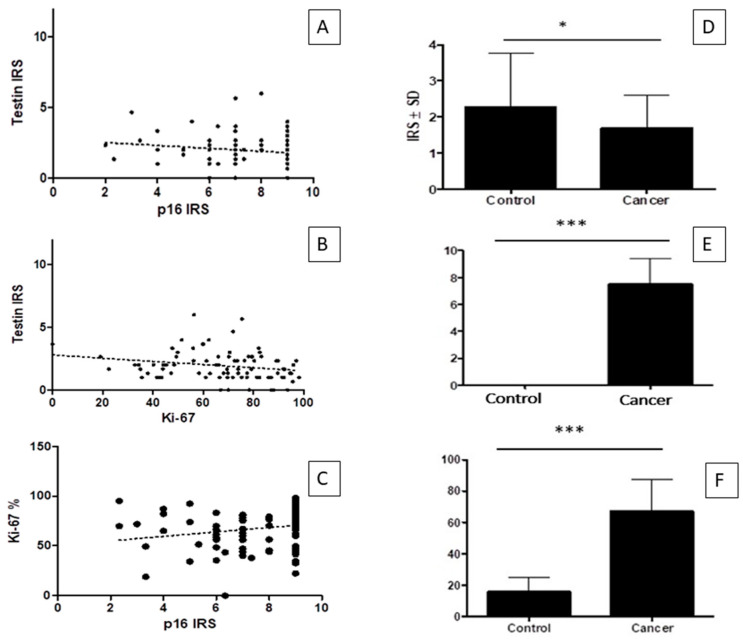
Immunohistochemical reactions in cervical cancer samples: (**A**) testin and p16 correlation in cervical cancer; (**B**) testin and Ki-67 correlation in cervical cancer; (**C**) Ki-67 and p16 correlation in cervical cancer; (**D**) testin expression in cervical cancer and control group; (**E**) p16 expression in cervical cancer and control group; (**F**) Ki-67 expression in cervical cancer and control group * *p* < 0.05 and *** *p* < 0.001.

**Figure 2:**
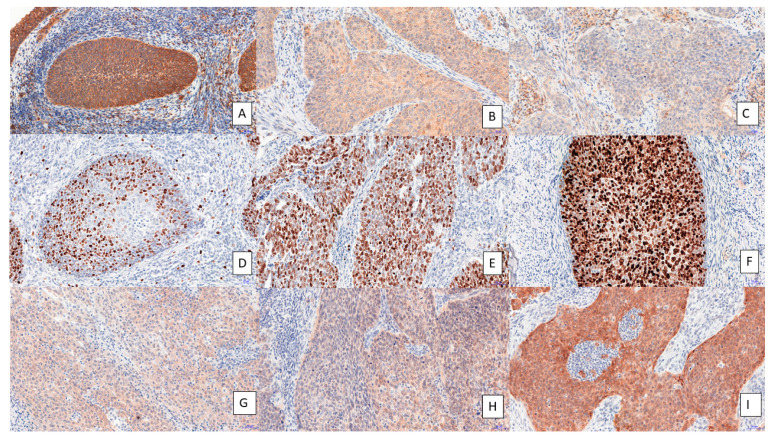
Immunohistochemical expression of testin in cervical cancer samples of different histological grades ((**A**), G1; (**B**), G2; (**C**), G3), Ki-67 ((**D**), G1; (**E**), G2; (**F**), G3) and p16 protein ((**G**), G1; (**H**), G2; (**I**), G3) in cervical cancer cases. Magnification ×200.

The immunohistochemical reactions for testin, p16 and Ki-67 expression were performed on all tissue specimens, i.e., 91 cervical cancer samples and 92 normal cervical tissue samples (Table 1). The histopathological analysis disclosed 73 cases of carcinoma planoepithelial, 12 cases of adenocarcinoma, 1 case of adenosquamous carcinoma and 5 cases of other histological types. The IHC analysis by Spearman correlation test showed that the expression of testin inversely correlated with the expression of p16 (r = −0.2104, *p* < 0.0465; Figure 1A) and Ki-67 (r = −0.2359, *p* = 0.0278; Figure 1B). Additionally, a positive correlation was observed between the expression of Ki-67 and p16 (*p* < 0.0082, r = 0.2819 Spearman correlation test; Figure 1C). The expressions of p16 and Ki-67 were significantly stronger in cancer cases than in the control group (*p* < 0.0001 for both; Mann–Whitney test; Figure 1E,F). In contrast, the expression of testin was significantly weaker in cancer cases than in the control group (*p* < 0.0113; Mann–Whitney test; Figure 1D). We observed a positive cytoplasmic reaction for testin (Figure 2A,C) and p16 protein (Figure 2G–I). The nuclear positive expression of Ki-67 correlated with the histological grade (Figure 2D–F).

### 3.2. WB on Cell Lines

**Figure 3:**
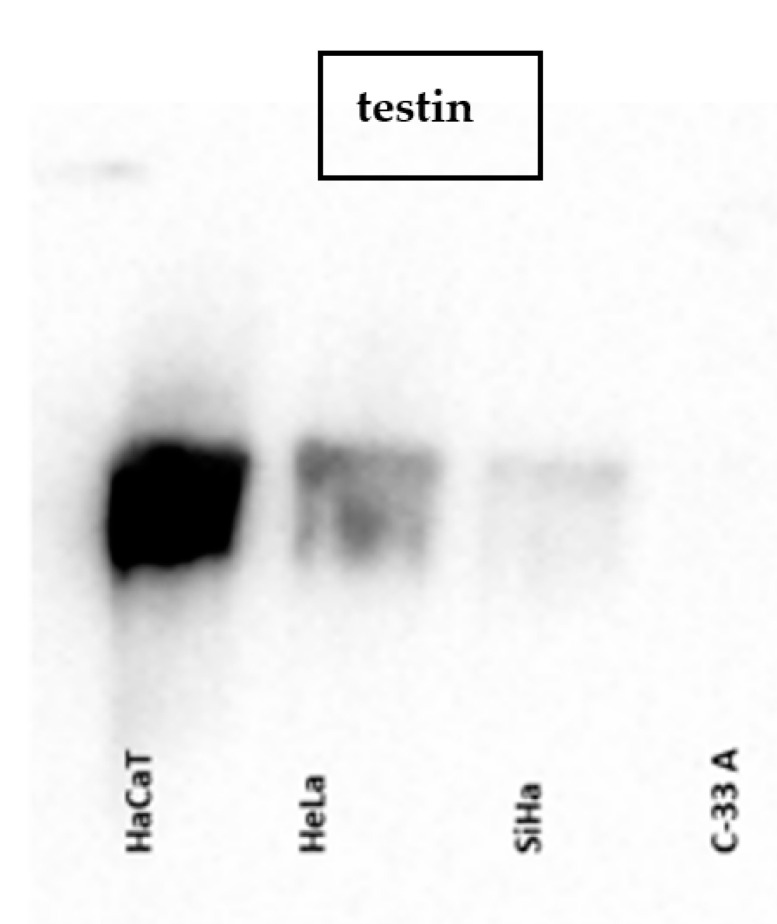
WB: testin expression in HaCat, HeLa, SiHa and C-33 A cell lines.

**Figure 4:**
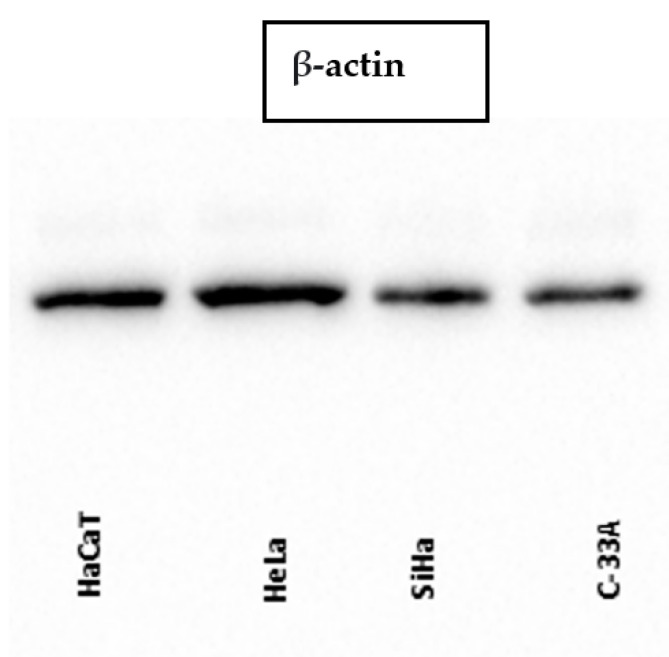
WB: β-actin expression in HaCat, HeLa, SiHa and C-33 A cell lines. The molecular weight of the proteins is: testin 54 kDa, β-actin 42 kDa.

**Figure 5:**
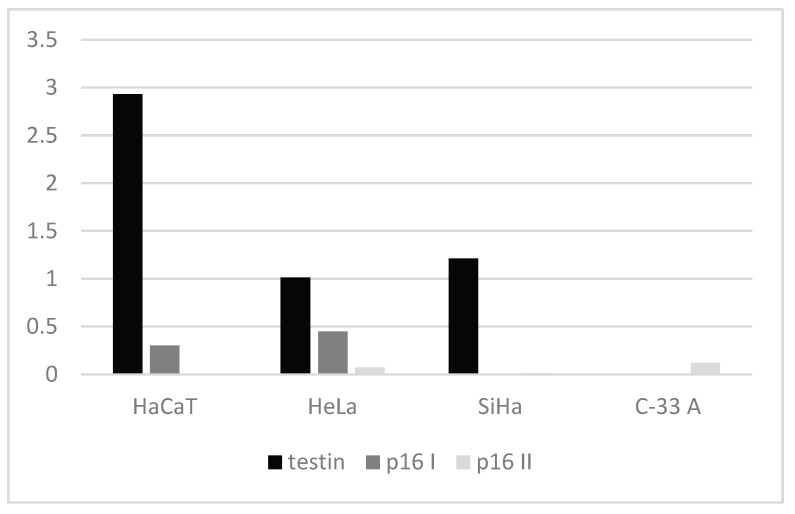
WB: testin and p16 expression in HaCat, HeLa, SiHa and C-33 A cell lines.

The analysis of testin expression was performed on the cervical cancer cell lines HeLa, SiHa (HPV positive) and C-33 A (HPV negative), as well on a human keratinocyte cell line (HaCaT) as a control. The WB analysis of testin expression in all cervical cancer cell lines corresponded to the IHC results and showed a weaker expression compared to that in the HaCaT cell line. When we compared the expression of testin in the cervical cancer cell lines, we found a weaker expression of testin in HPV-negative cell lines (Figure 3 and Figure 4). The expression of two isoforms of the p16 protein was lower in HaCaT, HeLa and SiHa cell lines compared to testin expression (Figure 5). The above results evaluated the presence of the protein within the cells. Table 3 present the quantification of the results of WB.

## 4. Discussion

Cervical cancer is one of the most malignant cancers among women. With a better understanding of the etiopathogenesis of this disease supported by appropriate screening, we may reduce the risk of death. Nowadays, cervical cancer screening is based on HPV testing and cytology. Depending on the local gynecological society recommendations, routine screening may vary in certain countries. Barely, these tests are based on age, belonging to a risk group or/and the HR-HPV status. In some cases, we expand the screening to clarify the diagnosis. For this reason, we need to perform histological additional testing. With the development of cervical cancer screening, immunohistochemical reactions in cytology has shown great promise for cervical screening [36,37]. Biomarkers which could provide detailed information about cancer prognosis and progression are in urgent need. In this study, we analyzed for the first time the relationship between widely used markers such as p16 and Ki-67 and testin. The low testin expression in cervical cancer cells is in line with that shown by Gu et al., who indicated in both in vitro and in vivo models the function of testin in cell proliferation and invasion in endometrial cancer [38]. Testin is expressed in almost all normal human tissues. We found various expression levels of the testin protein in cervical cancer-derived cell lines and compared it with the expression in human epidermal keratinocytes, i.e., the HaCaT cell line. Previously, Gu et al. showed that testin mRNA expression depends on the cervical cancer cell line. It was shown that testin suppressed cell proliferation through inhibiting cell cycle progression by arresting the cells in the G1 phase of the cell cycle [33]. Zhu et al. indicated the role of testin in cell migration and invasion and pointed out the protective capacity of testin in tumor metastasis and angiogenesis [38]. IHC expression analysis of p16 with Ki-67 is routinely performed in cervical cancer screening using commercial kits, e.g., CINtec, Dalton [37,39,40]. The expression of p16 slows the progression of the cell cycle from the G1 phase to the S-phase and inhibits the phosphorylation of the retinoblastoma protein (pRb) mediated through cyclin-dependent kinases [41]. Persistent HPV infection leads to p16 overexpression and consequently to Rb functionally inactivation by the HPV E7 oncoprotein [42,43]. In the present study, the correlation between testin and p16 expression was moderate and negative. However, there was a strong positive correlation between Ki-67 and p16 expression. The expression of p16 did not show any difference depending on the histological grade of cervical cancer. The combined overexpression of p16 and Ki-67 in normal cervical tissue is less likely to occur [37,44]. Thus, the analysis assessment of the expression of these markers is widely used in cervical cancer screening as a valuable tool for evaluating cell cycle deregulation and cell transformation due to HPV infection [45,46]. Ki-67 is a marker strictly associated with cell proliferation, expressed in all active phases of the cell cycle (G1, S, G2, M) except the G0 phase. In normal cervical tissue, the expression of Ki-67 is restricted to one-third of the basal layer of the epithelium but in dysplasia and cancer, it is expanded above the basal and parabasal layers, and the number of positive cells increases [47]. Our study disclosed that Ki-67 overexpression was present in 100% of invasive cervical cancer samples. This is concordant with Shi et al. study [42]. The positivity rate of Ki-67 expression was significantly higher in cervical cancer cells than in control cells.

### Limitations

Our study has some limitations. The major limitation is the small study sample. Moreover, the number of analytical methods used (IHC and WB) was limited; however, additional techniques such as at least reverse transcription–polymerase chain reaction (RT-PCR) are planned in the future.

## 5. Conclusions

In summary, we found that the intensity of testin expression and the number of positive cells reversely correlated with the expression of Ki-67 (a marker of proliferation) and p16 (a marker of cell cycle dysregulation). These three markers may complement each other, and their combined assessment may improve cervical cancer diagnostics. As in this study protein expression was presented, gene expression remains to be explored. Not-withstanding and unquestionably, further studies are necessary to discuss the above-presented results in the broadest context as possible.

## Figures and Tables

**Table 1:** Characterization of the study group and histological types of the cervical cancer samples.

Parameters	No.	(%)
**Age (years)** <35	7	7.69
35–45	22	24.18
>45	62	68.13
**Grade** G1	18	19.78
G2	57	62.64
G3	16	17.58
**Histological types** **Carcinoma planoepithelial**	73	80.22
Adenocarcinoma	12	13.19
Other than carcinoma planoepithelial and adenocarcinoma	6	6.59

**Table 2:** Scoring system by Remmele and Stegner (IRS, Immunoreactive Score) taking into account the percentage of cells and the intensity of the reaction.

Score	Percentage of Positively Stained Cells (PP)	Intensity of Staining (SI)	IRS Points (PP × SI)	IRS Classification
**0**	no staining	no color reaction	0–1	Negative
**1**	<10%	weak reaction	2–3	Positive, weak expression
**2**	10–50%	moderate reaction	4–8	Positive, moderate expression
**3**	51–80%	strong reaction	9–12	Positive, strong expression
**4**	>80%			

**Table 3:** Quantification of the WB bands for testin and β-actin.

	Control			Testin			
	β-Actin I	β-Actin II	β-Actin Average	Testin I	Testin II	Testin Average	Testin Normallized
**HaCaT**	2,179,147	1,980,546	2,079,847	6,246,846	5,946,842	6,096,844	2.931391331
**HeLa**	2,362,978	1,897,653	2,130,316	2,209,494	2,106,849	2,158,171.5	1.013075997
**SiHa**	2,008,224	2,012,950	2,010,587	471,170	4,405,306	2,438,238	1.212699575
**C-33A**	1,989,734	2,106,843	2,048,289	12,936	10,310	11,623	0.005674494

## Data Availability

The data presented in this study are available on request from the corresponding author. The data are not publicly available due to privacy issues.
